# An efficient, reliable and valid assessment for affective states during online learning

**DOI:** 10.1038/s41598-024-66974-2

**Published:** 2024-07-09

**Authors:** Oi-ling Siu, Kelvin F. H. Lui, Yi Huang, Ting Kin Ng, Wai Lan Victoria Yeung

**Affiliations:** 1 Department of Psychology, Lingnan University, Tuen Mun, Hong Kong, China; 2 Wofoo Joseph Lee Consulting and Counselling Psychology Research Centre, Lingnan University, Hong Kong, China

**Keywords:** Affective states, Online learning, Assessment tool, Learning performance, Validity, Psychology, Human behaviour

## Abstract

The current study aims to develop an efficient, reliable and valid assessment, the affective states for online learning scale (ASOLS), for measuring learners’ affective states during online learning using a sample of 173 young learners. The assessment consists of 15 items which assess five affective states, including concentration, motivation, perseverance, engagement, and self-initiative. To improve efficiency, five items (one for each affective state) are randomly selected and presented every 30 min during online learning. In addition, 14 among the participants were further invited to perform on-site online learning, and their affective states were validated by observations conducted by two psychologists. The ASOLS was found to be reliable and valid, with high internal consistency reliabilities and good construct, convergent and criterion validity. Confirmatory factor analyses showed that the hypothesized five-factor structure demonstrated a satisfactory fit to the data. Moreover, engagement was found to be positively associated with learning performance. Our findings suggest that the ASOLS provides a useful tool for teachers to identify students in upper primary and junior secondary schools with deficits in affective states and offer appropriate remedy or support. It can also be used to evaluate the effectiveness of interventions aimed at enhancing students’ affective states during online learning.

## Introduction

### The prevalence of online learning

Online learning is defined as learning with electronic devices with internet access in which the learner can learn independently or interactively with the instructors and other learners^1^. Decades ago, online education and learning had already emerged in many schools and universities around the world, which allows students to learn at their homes with internet access^2^. The more recent rapid development of technologies has facilitated online education and learning and made it more convenient, prevalent and popular^3^. During the most serious periods of the Covid-19 pandemic, online education and learning even became the only option in many cities around the world^4^. For example, all students in Hong Kong, including primary school students, secondary school students, and university students, engaged in online learning during the quarantine of Covid-19. Although it was suggested that the rapid shift in teaching mode during the pandemic should be a type of ‘emergency remote teaching’ which is different from well-planned online education, findings indicated a notable increase in various online learning activities in the post-COVID era^5^.

Previous research has indicated that online learning is especially suitable for gifted students^6,7^. Gifted students are defined as students with outstanding performance or potential in areas such as intelligence, specific academic aptitude, creativity, leadership, visual or performing arts, or psychomotor abilities^8^. Gifted students often experience boredom in mainstream classrooms as the general curriculum are not challenging to them^9^. Online learning can meet gifted students’ needs by offering them opportunities to access advanced curricula that are unavailable in regular schools^6,7^.

### The importance of efficient assessments for affective states during online learning

Despite the prevalence and popularity of online education and learning in the modern world, there are various problems associated with online learning. One particular concern is about students’ affective states which were found to be associated with the outcomes of online learning^10^. Therefore, it is important to assess and monitor students’ affective states during online learning. However, when students are engaged in online learning independently, no instructors can monitor their affective states and provide feedback and instructions on learning to them. Even during interactive online learning with an instructor and other students, it is usually difficult for the instructor to monitor all the students’ affective states through the computer screen during the teaching.

The influences of affective states on online learning outcomes may be more pronounced for gifted students. While gifted students are expected to be outstanding learners, many gifted students fail to realize their academic potential^11^. Scholars have posited that the underachievement of gifted students is often caused by affective factors such as low levels of concentration^12^, motivation^11^, perseverance^13^, engagement^14^, and self-initiative^15^. It is important to understand gifted students’ affective states during online learning in order to provide them with suitable support and guidance. As a result, an efficient, reliable, and valid assessment tool is necessary for measuring the affective states during online learning among gifted students.

### Affective states affecting online learning outcomes

In the literature, a large set of affective states have been studied. For example, a previous study identified 17 affective states and suggested that flow/engagement, confusion, and boredom were the most frequent affective states experienced by students during individual learning^16^. In the current study, we focused on a smaller set of affective states (i.e., concentration, motivation, perseverance, engagement, and self-initiative) that are representative and important for the outcomes of online learning. Among the larger set of affective states, many emotions were found to be highly associated with these five affective states. For instance, a study found that frustration, confusion, and boredom showed moderate to large correlations with engaged concentration, *r* = − 0.76, − 0.4, and − 0.36, respectively^17^. In addition, another study reviewed many studies and suggested relationships between pleasant and unpleasant affects (e.g., happiness and sadness) and motivation, persistence (i.e., perseverance), engagement, and self-regulated learning (i.e., self-initiative)^18^. More recently, a study conducted a Strengths, Weaknesses, Opportunities, & Challenges (SWOC) analysis of online learning on secondary data gathered from various sources including journals, research articles, search engines, and company websites. The SWOC analysis found that students’ nonserious learning behaviours was one of the major weaknesses of online learning^4^. As students may find online learning boring, lacking community, and difficult to understand the instructional goals, they may show various affective problems during online learning such as lack of attention, low motivation, and unengaging behaviours, which may in turn affect their learning outcomes. The importance of each of these five affective states on the learners’ online learning outcomes is briefly reviewed below. A more comprehensive and systematic review is included in Supplementary Materials I.

#### Concentration

Human attention is a limited cognitive resource. Dividing attention between two tasks can lead to performance detriment in both tasks^19,20^. This is also because people cannot perform some cognitive processes concurrently for more than one task such as retrieving the task information from long-term memory, reconfiguring the cognitive system for a new task, and selecting the appropriate task response^21^. However, due to the rapid development of technologies and media, it has become very prevalent for teenagers and students to engage in media multitasking in daily life^22,23^. Meanwhile, many studies found that engaging in multitasking behaviours or being less concentrative during learning impaired the learning outcomes^24–27^. Therefore, students’ concentration level is one of the most important affective states we need to assess and monitor during their online learning.

#### Intrinsic motivation

Students’ motivation in learning is usually divided into internal/intrinsic and external/extrinsic motivation^28^. Internal motivation comes from a student’s interest in the task itself, such as curiosity about something^29^. External motivation refers to a student’s involvement in learning due to some external incentive, such as getting a high grade, avoiding punishment, or competition^30^. A study conducted a large-scale meta-analysis on 344 samples (223,209 participants) and found that students’ intrinsic motivation was the key factor for their academic achievement and well-being, while extrinsic motivation was only partly associated with academic achievement but negatively associated with well-being^30^. Research on online learning environments also suggested that the learner’s motivation was a very important factor for the success of an online learning experience^31–33^. Therefore, the current study focused on the students’ intrinsic motivation as another affective state affecting their online learning outcomes.

#### Perseverance

Perseverance is defined as the capacity to pursue one’s goals till completion even when encountering difficulties^34^. In the context of student learning, perseverance refers to persistence in learning and completion of the learning tasks^35^. In a study, observed persistence was a key predictor of children’s learning-related behaviours and academic achievement^35^. In a subsequent study, it was found that high-perseverance students were able to solve more difficult tasks than low-perseverance students in a digital educational game about history^36^. In a more recent study, the authors examined the mechanisms underlying the positive association between perseverance and academic achievement and found that perseverance improved academic achievement through improving self-regulated learning and motivation^37^. Perseverance was also found to be moderately correlated with engagement, which may also explain its positive association with academic achievement^34^.

#### Engagement

Study engagement is a concept modelled after work engagement as students’ study and learning can be considered as their ‘work’^38,39^. Engagement in the current study is conceptualized as a positive, fulfilling state characterized by vigour, dedication, and absorption modelled from a previous work in university students^40^. Vigour refers to feeling energetic, resilient, and eager to work; dedication refers to devoting oneself to work with high emotional arousal; and absorption refers to totally immersing oneself in work and forming a deep connection with work while feeling detached from other things. Many studies have shown that engagement was positively associated with learning outcomes^38,39,41^.

#### Self-initiative

Self-initiative is defined as intentional goal-directed behaviours to achieve success^42^. In the context of learning, it refers to self-directed learning in which the students take the initiative to manage their own learning processes such as identifying the learning needs and goals, planning the learning activities, searching for the learning materials and resources, and implementing the learning activities^43^. A recent study suggested that students with high self-directed learning ability engaged in significantly more planning for learning behaviours and demonstrated significantly more reading outcomes than students with low self-directed learning ability^44^. In a laboratory experimental study, self-direct learning ability was also found to be positively associated with online learning performance in engineering students^45^. Self-directed learning has been suggested to be particularly important for online learning as online learning itself is a self-directed learning experience in which the learners have to take control in planning, monitoring, and making decisions for their learning processes^46^. They may also need to actively explore various learning resources in the online learning environment and develop strategies to effectively use the resources to maximize the learning outcomes.

### Existing affective states assessments

As reviewed above, the affective states during learning including concentration, motivation, perseverance, engagement, and self-initiative, are important for the learning outcomes, particularly for online learning in which students have more control over their learning processes and activities. Therefore, an efficient, reliable, and valid online assessment which can assess students’ affective states and then provide them with feedback to maintain their positive affects during online learning is crucial for their online learning success. The existing measurements for affective states are mostly self-reported questionnaires for a single affective state such as the Multitasking Preference Inventory^47^, the Intrinsic Motivation scale^40^, the Study Engagement Scale^40^, and the Self-directed Learning Readiness Scale^43^. Many of the measurements were not specifically designed to be used in online learning for young learners and some measurements such as the Multitasking Preference Inventory were even not originally designed to be used in the learning context.

There were also physiological and neuroimaging measurements for the affective states. For example, several previous studies assessed the emotional states by measuring the heart rate variability (HRV) with an ear sensor^48,49^. In addition, concentration in learning can be assessed by measuring the temperature and pulse on the fingertips^50^, by measuring the electroencephalography (EEG)^49,51^, and by hybrid methods combining head pose and eye tracking detection^52^. However, these physiological and neuroimaging measurements are hard to implement by the learners themselves during online learning. Another study used the length of time spent on a video to indicate the degree of study engagement^53^. However, as noted by the authors, a limitation of this behavioural indicator was that it could not tell whether the learner is actively paying attention to the video or just playing the video in the background while multitasking. To conclude, there is a need to develop an efficient, reliable, and easy-to-use measurement for learners or instructors to monitor the learners’ affective states during online learning.

### The present study

The objective of the current study is to develop an efficient and valid assessment for affective states of young learners during online learning. The assessment would differ from most of the existing affective state assessments in the following aspects. Firstly, it should be sufficiently short to be completed within a few minutes so that the completion of the assessment will not disturb the learner’s online learning process. Secondly, the assessment should cover the various aspects of affective states which are highly associated with the learning outcomes. Finally, the assessment should also be easy to use so that the learners and instructors can implement the assessment themselves and use the results to construct feedback for the learners to maintain the positive affects during online learning. The present study developed a short self-reported assessment with 15 items, namely the affective states for online learning scale (ASOLS), for measuring five affective states including concentration, motivation, perseverance, engagement, and self-initiative when a learner is engaging in online learning. The reliability and validity of this efficient assessment were evaluated. Confirmatory factor analyses (CFAs) were also conducted to examine the factor structure of the ASOLS.

## Results

### Descriptive statistics and reliabilities

Table 1 shows the descriptive statistics and reliabilities of the affective states measured by the ASOLS (i.e., during the online learning at home) and evaluated by the psychologists (i.e., during the on-site learning at the ASTRI). The means of the affective states measured by the ASOLS were around 5. For the on-site learning evaluation, the means of the affective states were around 4 which were slightly lower. The standard deviations were comparable across the two assessment methods with most of the values around 1. The ranges of the ratings were large across students, suggesting that individual differences were observed among students.

**Table 1:** Descriptive statistics and reliabilities of the affective states measured by the two assessment methods.

	*M*	*SD*	Range	α	ω
ASOLS					
Concentration	4.90	0.96	1.0–6.0	0.893	0.897
Motivation	5.04	0.98	1.0–6.0	0.939	0.940
Perseverance	5.09	0.89	1.0–6.0	0.916	0.921
Engagement	4.56	1.11	1.0–6.0	0.892	0.894
Self-initiative	5.06	0.97	1.0–6.0	0.951	0.952
On-site observation	*M*	*SD*	Range	ICC	
Concentration	4.03	1.26	2.5–6.0	0.816	
Motivation	4.00	1.09	2.0–5.5	0.865	
Perseverance	4.27	1.28	2.0–6.0	0.869	
Engagement	4.10	1.12	2.17–5.67	0.875	

Reliabilities of the ASOLS were assessed by two internal consistency reliabilities including the Cronbach’s α and McDonald’s ω coefficients. As shown in Table 1, the reliabilities of the ASOLS were high for all the 5 affective states. The Cronbach’s α coefficients ranged from 0.892 to 0.951, and the McDonald’s ω coefficients ranged from 0.894 to 0.952, indicating adequate internal consistency reliability. For on-site evaluation, the inter-rater reliabilities of the two observers’ ratings were assessed by the intraclass correlations (ICCs). The inter-rater reliabilities were high for all the 4 affective states. The ICCs were 0.816, 0.865, 0.869, and 0.875 for concentration, motivation, perseverance, and engagement, respectively.

### Confirmatory factor analyses

To examine the factor structure of the ASOLS, LISREL 8.80 was utilized to conduct CFAs. In addition to the hypothesized five-factor model, two alternative models were also tested. In particular, the following models were tested: (a) a one-factor model, (b) the hypothesized five-factor model, and (c) a hierarchical model in which five first-order factors were loaded on a second-order general factor.

Given that the multivariate skewness and kurtosis tests revealed that the data did not adhere to multivariate normality (*p*s < 0.001), it was inappropriate to utilize maximum likelihood (ML) estimation. Instead, the robust maximum likelihood (RML) estimation method was utilized, and the Satorra–Bentler scaled χ2 (S-Bχ2) statistic was computed to adjust for non-normality^54^. The fit of the models was evaluated using various indices, including the root mean square error of approximation (RMSEA)^55^, the comparative fit index (CFI)^56^, standardized root mean squared Residual (SRMR), and Tucker–Lewis index (TLI)^57,58^. Also, an RMSEA ≤ 0.10 indicates an acceptable fit, while ≤ 0.08 suggests an adequate fit^59^. Additionally, a CFI ≥ 0.95, a TLI ≥ 0.95, and an SRMR ≤ 0.08 generally indicate a good model–data fit^60^. For model comparison, the Akaike information criterion (AIC)^61^ was used since both nested and non-nested models were considered. A lower AIC value indicates a better fit for the model.

**Figure 1:**
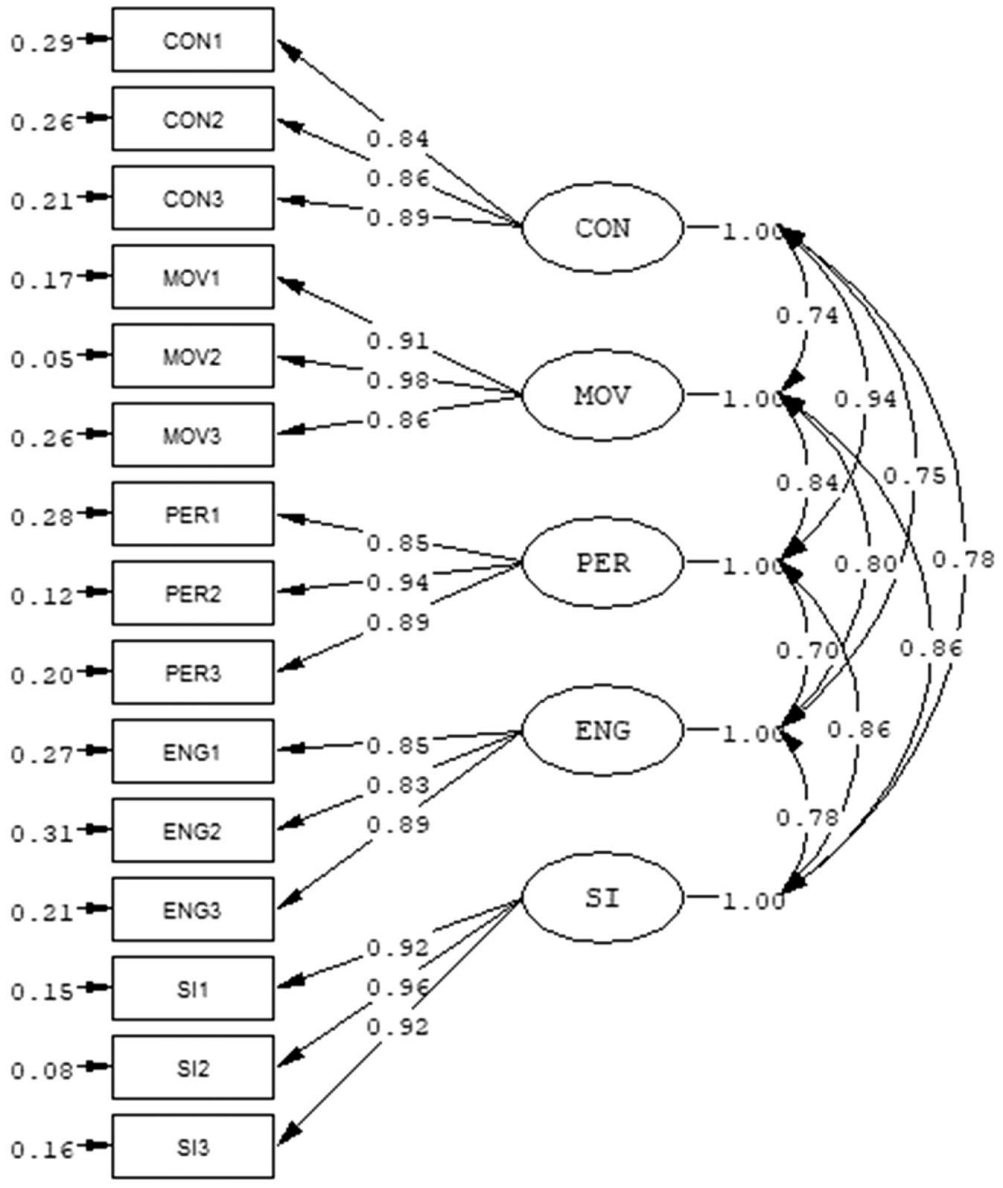
Model diagram for the hypothesized five-factor model for the Affective States for Online Learning Scale. The hypothesized five-factor model for the Affective States for Online Learning Scale. *CON* Concentration, *MOV* Motivation, *PER* Perseverance, *ENG* Engagement, *SI* Self-initiative. Standardized coefficients are reported. All factor loadings and factor correlations are significantly at *p* < .001.

Table 2 summarizes the findings of the CFAs. The one-factor model did not adequately fit the data, while both the five-factor model and the hierarchical model showed a good fit. The AIC value for the five-factor model was the smallest among the three models, indicating that it was the best fitting model, S-Bχ^2^(80) = 165.99, *p* < 0.001, RMSEA = 0.08, 90% CI [0.06, 0.10], CFI = 0.99, TLI = 0.98, SRMR = 0.043, AIC = 229.99. These results supported the hypothesized five-factor model. Figure 1 displays the coefficients of the model, with all factor loadings > 0.30 (*p*s < 0.001). Additionally, the five factors were significantly correlated with each other (*r* = 0.70 to 0.94, *p*s < 0.001).

**Table 2:** Confirmatory factor analyses of the affective states for online learning scale.

Model	S-Bχ^2^	*df*	CFI	TLI	SRMR	RMSEA [90% CI]	AIC
1. One-factor model	230.80***	90	0.98	0.97	0.072	0.11 [0.09, 0.13]	290.80
2. Five-factor model	149.99***	80	0.99	0.98	0.043	0.08 [0.06, 0.10]	229.99
3. Hierarchical model	165.99***	85	0.99	0.98	0.057	0.09 [0.07, 0.11]	235.99

***p < .001.

### Correlations between the two assessment methods

The convergent validity of the ASOLS was first evaluated by examining the correlations of ratings of the same affective state between the ASOLS and the on-site observation across the 14 students who attended the on-site learning. As shown in Table 3, the correlations between the two assessment methods were statistically significant, *r*s = 0.518 (*p* = 0.058), 0.584 (*p* = 0.028), 0.592 (*p* = 0.026), and 0.579 (*p* = 0.030) for concentration, motivation, perseverance, and engagement, respectively. The correlations were medium to large, suggesting that the convergent validity of the ASOLS was satisfactory. This was particularly promising given the small sample size and the large discrepancy in the nature between the two assessment methods (i.e., self-report questionnaire vs. behavioural observation). To conclude, the results of the ASOLS were in consistent with that of the on-site observation, suggesting that the convergent validity of the ASOLS was good.

**Table 3:** Correlations among the affective states of the ASOLS and on-site observation.

		ASOLS					On-site observation			
		Concentration	Motivation	Perseverance	Engagement	Self-initiative	Concentration	Motivation	Perseverance	Engagement
ASOLS	Concentration	–	0.680***	0.871***	0.673***	0.736***	0.518	0.568*	0.591*	0.598*
	Motivation		–	0.765***	0.748***	0.809***	0.314	0.584*	0.594*	0.527
	Perseverance			–	0.643***	0.814***	0.418	0.581*	0.592*	0.564*
	Engagement				–	0.736***	0.470	0.603*	0.559*	0.579*
	Self-initiative					–	0.413	0.608*	0.499	0.536*

* < .05; ** < .01; *** < .001.

### Correlations among the affective states

As shown in Table3, the correlations among the affective states were very high in general (i.e., all larger than 0.600 and 13 out of 16 correlations larger than 0.700). Apparently, high correlations among the subscales (i.e., affective states) may suggest low discriminatory validity of the ASOLS; however, this was probably because the five constructs of the affective states were theoretically related among themselves. For example, a highly motivated student may be more concentrated and engaged in learning. A student with an intention to succeed in learning (i.e., high self-initiative) may be more likely to pursue his or her learning goals till completion even when encountering difficulties (i.e., high perseverance). High correlations among the affective states also provided evidence for the construct validities of the ASOLS.

### Associations with the learning performance

The criterion validity of the ASOLS was evaluated by examining their associations with the learning performance of the online course. A hierarchical linear regression was performed with the online course examination score as the dependent variable, age, gender, father’s education, and mother’s education as the controlled variables (i.e., entered in block 1), and the five affective states as the independent variables (i.e., entered in block 2). Students who did not complete the course (i.e., no exam scores) were excluded from the analysis. Overall, the predictors explained a 20.9% variance in learning performance. As shown in Table 4, engagement was significantly and positively associated with learning performance, β = 0.389, *t* = 2.02, *p* = 0.048, suggesting that students with a higher level of engagement performed better in the examination of the online course. The other four affective states were not significantly associated with learning performance.

**Table 4:** Results of the hierarchical linear regression.

Variables	Online course examination score				
	*B*	*SE*	β	*t*-score	*p*-value
Age	0.003	0.009	0.042	0.302	0.763
Gender	− 0.012	0.030	− 0.050	− 0.395	0.694
Father’s education	0.011	0.045	0.047	0.249	0.804
Mother’s education	0.054	0.042	0.222	1.29	0.204
Concentration	0.006	0.030	0.057	0.210	0.835
Motivation	− 0.058	0.031	− 0.503	− 1.90	0.063
Perseverance	0.000	0.042	0.002	0.006	0.995
Engagement	0.039	0.019	0.389	2.02	0.048*
Self-initiative	0.014	0.029	0.124	0.484	0.630

* < 0.05. Engagement was significantly and positively associated with the learning performance, *t* = 2.02, *p* = 0.048, suggesting that students with a higher level of engagement performed better in the examination of the online course.

## Discussion

The current study aims to develop an efficient, reliable, and valid assessment for measuring the learners’ affective states during online learning. The affective states assessment developed in the current study, the ASOLS, contained 15 items assessing five affective states including concentration, motivation, perseverance, engagement, and self-initiative. To ensure the efficiency of the ASOLS, in the design of the assessment, five items (one for each affective state) from the assessment are randomly selected and popped up every 30 min for the learners to answer before they can continue the online learning. As the learners just need a minimal amount of time to complete the five assessment items, the interruption of the online learning caused by this efficient assessment is minimized. As a result, the ASOLS can measure the learner’s affective states efficiently and effectively without disturbing the learner’s online learning process.

The reliability of the ASOLS was very good. As the participants completed five items once every 30 min, most of them completed the assessment multiple times. We examined the internal consistency of the assessment for all the 5 affective states items by averaging the assessment trials for the same items and then calculating the Cronbach’s α and McDonald’s ω coefficients among the three items for each affective state. The Cronbach’s α and McDonald’s ω coefficients were very high (i.e., *r*s > 0.892). This suggests that the items measuring the same affective states produced highly consistent results among themselves. In other words, the ASOLS is not only efficient but also reliable.

The validity of the ASOLS was evaluated by examining the construct validity, convergent validity and criterion validity. The results of the confirmatory factor analysis provided support for the suggested five-factor framework, demonstrating a satisfactory fit for ASOLS and providing good support for the construct validity of the assessment. To examine the convergent validity, we invited 14 participants to participate in a 2-h online session in the ASTRI in which their learning behaviours and affective states were observed and evaluated by two psychologists. This on-site observation method for the affective states showed good inter-rater reliabilities (i.e., ICCs > 0.816). More importantly, the correlations between the affective state ratings of the ASOLS and on-site observation were moderate and significant (*r*s > 0.518) across the 14 participants, suggesting a good convergent validity of the ASOLS. This was particularly promising given the small sample size and the large discrepancy in the nature between the two assessment methods (i.e., self-report questionnaire vs. behavioural observation). For the criterion validity, we examined the association between the affective states measured by the ASOLS and the students’ learning performance. The online course contained a final exam which the participants were required to take prior to the completion of the course. A hierarchical linear regression was performed to examine the associations between the affective states and the learning performance after controlling for the students’ age, gender, and their parents’ education level. Engagement was found to be positively associated with learning performance. This was consistent with the previous findings showing a positive association between study engagement and learning outcomes^38,39,41^. However, the other four affective states were not significantly associated with learning performance which was not consistent with the previous findings suggesting a positive association between the affective states and learning performance^27,32,35,45^. This is probably because, compared to other affective states, engagement is a more comprehensive affective state which reflects not only a student’s intrinsic motivation, but also his/her emotional arousal during the study and behavioural immersion in learning. It is reasonable that engagement showed the largest unique contribution to learning performance after controlling for other affective states. To conclude, the result suggested that the ASOLS had good construct validity, convergent validity and criterion validity.

### Practical implications

This study has important practical implications. The ASOLS provides a useful tool for teachers to understand students’ levels of affective states that are crucial to the effectiveness of online learning. Teachers can employ this instrument to identify students with deficits in concentration, motivation, perseverance, engagement, or self-initiative during online learning and provide them with appropriate support. Furthermore, this instrument can be utilized multiple times across sessions to detect changes in affective states for evaluating the efficacy of interventions that aim at enhancing students’ affective states during online learning. For example, a recent study developed an online intervention targeting students' intrinsic motivation for online learning tasks^62^. The motivation subscale of our instrument can be used to assess students’ improvement in intrinsic motivation for online learning following the intervention.

### Limitations and future directions

All of the participants in the present study are gifted students. Therefore, it is not completely clear whether the findings of the present study can be generalized to typically developing students and other populations. Future studies should further validate the ASOLS on typically developing students and other populations such as learners of other age groups. Due to the pandemic, we only collected a relatively small sample size. The small sample size for the on-site learning evaluation session is also a limitation. Future studies should recruit a larger sample size to enhance the evidence of convergent validity.

Besides, the current study only measured the learners’ affective states during online learning but did not give them feedback to facilitate their learning. Future research should implement an automatic scoring algorithm for the affective state ratings, provide feedback to the learners immediately after they have completed the assessment based on the ratings, and evaluate the effectiveness of the feedback in facilitating their learning. For example, if a learner reports that he or she did not engage in and concentrate on learning, we may remind him or her to be more concentrated or to take a break if he or she is too tired to learn. Previous research suggested that taking a break during learning will not harm the learning outcomes^36^. The ASOLS should incorporate these findings to provide constructive feedback messages to the learners to facilitate their learning. If this is possible, this efficient assessment tool will improve the learning outcomes without causing any disruption to the learning process.

## Conclusion

Due to the prevalence of online education and learning in the modern world and the various problems associated with online learning, it is imperative to develop an efficient, reliable, and valid assessment for affective states during online learning. The ASOLS is efficient and was shown to be reliable and valid in assessing the affective states of young gifted students during online learning. This efficient, reliable, and valid affective states measurement has the great potential to be used in monitoring learners’ affective states during self-learning and even provide feedback for them to facilitate their learning outcomes.

## Method

### Participants

Participants were gifted students recruited from The Hong Kong Academy for Gifted Education who attended two online courses including a course about earth science and another course about paleontology. We aimed at recruiting as many students as possible. The final sample included 173 gifted students (84 male) with 65 students learning earth science and 109 students learning paleontology. Participants were either primary school or junior secondary school students with an average age of 10.63 years old (age range: 9–17, *SD* = 1.74). Among the 173 students, 14 of them (male: *N* = 7, female: *N* = 7) were invited to participate in an on-site learning evaluation session. They were on average 11 years old (age range: 9–14, *SD* = 1.36). Research ethics was approved by the Research Committee of Lingnan University (Ref. no. EC106/2122). All procedures performed in this study were in accordance with the ethical standards of the institutional and/or national research committee and with the 1964 Helsinki Declaration and its later amendments or comparable ethical standards. An informed consent was obtained from the parents of the students.

### Procedure

Both quantitative (self-report survey) and qualitative approaches (classroom observations) were adopted. An affective states assessment was developed to assess a learner’s five affective states, including concentration, motivation, perseverance, engagement, and self-initiative during online learning. The assessment was administered to 173 gifted students who attended two online courses in The Hong Kong Academy for Gifted Education. Among the 173 students, 14 of them were also invited to participate in an on-site learning evaluation session in which the five affective states of these students were also evaluated by two psychologists through observations. The reliability of the assessment was evaluated by examining the internal consistency reliabilities including the Cronbach’s α and the McDonald’s ω coefficients. The validity of the assessment was evaluated by examining the construct validity (i.e., performing a confirmatory factor analyses to validate the factor structure of the assessment), convergent validity (i.e., examining associations with the on-site evaluation results provided by the two psychologists) and the criterion validity (i.e., examining associations with the learning outcomes of the online courses).

### The affective states for online learning scale

The affective states for online learning scale (ASOLS) contained 15 items assessing the five affective states with 3 items for each affective state. All items were rated on a 6-point Likert scale (i.e., from 1 = strongly disagree to 6 = strongly agree) according to the learning experience in the past half an hour. The Chinese and English versions of the items are shown in Supplementary Materials II. The Chinese version was used in the current study. Details of the items for each affective state were described below.

#### Concentration

Concentration is defined as the proportion of mental resources and attention allocated to the learning tasks while engaging in learning. The three concentration items were adapted from the Item 8, 13, and 14 of the Multitasking Preference Inventory^47^, which is a measurement for polychronicity reflecting the preference for multitasking (i.e., allocating attention to more than one task) as opposed to performing only a single task (i.e., high concentration) at a time. The items were translated to Chinese and revised briefly to better measure the participants’ concentration during online learning.

#### Motivation

Motivation is usually divided into internal/intrinsic and external/extrinsic motivation^28^. Intrinsic motivation comes from a student's interest in the task itself, such as curiosity about something and was the focus of this assessment^29^. The three intrinsic motivation items were adapted from the Item 1, 8, 14 of the Intrinsic Motivation scale^40^.

#### Perseverance

Perseverance is defined as the capacity to pursue one’s goals till completion even when encountering difficulties^34^. In the context of student learning, perseverance refers to the persistence in learning and completion of the learning tasks^35^. The three perseverance items were adapted from the perseverance subscale (P1, P2, P3) of the Chinese EPOCH Measure which was a psychometrically sound measure of perseverance among Chinese students aged from 6 to 19 years^34^. Three items (P1, P2, P3) from the perseverance subscale of the Chinese EPOCH Measure were adapted to measure perseverance in online learning^34^.

#### Engagement

Study engagement is defined as a state characterized by vigour, dedication, and absorption^40^. Vigour refers to feeling energetic, resilient, and eager to work; dedication refers to devoting oneself to work with high emotional arousal; and absorption refers to totally immersing oneself in work and forming a deep connection with work while feeling detached from other things. The three engagement items were adapted from the absorption subscale of the short Chinese version of the Study Engagement Scale^40^.

#### Self-initiative

Self-initiative is defined as intentional behaviours conducted by the students to achieve success in learning activities^42^. In the context of learning, it refers to self-directed learning in which the students take the initiative to manage their own learning processes. The three self-initiative items were adapted from the desire for learning subscale of the Self-directed Learning Readiness Scale^43^. The items were translated to Chinese and revised briefly to better measure the participants’ self-initiative during online learning.

### Assessment for affective states during online learning at home

All participants attended a well-designed online course, either in the subject of earth science or paleontology, at home through an online platform. They were allowed to log into the platform to pursue online self-learning anytime during the learning period. The learning pace and progress were fully controlled by the participants themselves. During the online learning, three different sets of five items (one for each affective state) of the ASOLS popped up every 30 min. The participants were required to answer all of the five items in order to continue the online learning. Each participant’s responses to items of the same affective state were averaged, giving a composite score for each affective state. The composite scores ranged from 1 to 6. A higher composite score indicates a higher level of concentration, motivation, perseverance, engagement, or self-initiative during online learning.

### Assessment for affective states during on-site learning

Among the 173 participants, 14 of them were invited to participate in a 2-h online learning session at the Hong Kong Applied Science and Technology Research Institute Company Limited (ASTRI). Each session had no more than three students. Two psychologists kept around a 2-m distance from the students and sat on the opposite side of the students to observe and evaluate the students’ affective states based on their overt learning behaviours. Four affective states (concentration, motivation, perseverance, and engagement) were evaluated in the on-site learning evaluation sessions, as self-initiative cannot be easily observed in a 2-h self-learning session. The final rating of each affective state was scored using a 6-point Likert scale. A higher rating indicates a higher level of concentration, motivation, perseverance, and engagement during online learning.

### Learning outcomes

The online course contained a final examination which the students were required to take prior to the completion of the course. The examination score was recorded and used as an indicator of learning performance.

## Open practices statement

This study was not preregistered.

## Supplementary Information

Supplementary Information.

## Data Availability

The data sets analyzed during the current study are available from the corresponding author on reasonable request. The assessment items were attached as Supplementary Materials of this manuscript.
