# Author Correction: An efficient, reliable and valid assessment for affective states during online learning

**DOI:** 10.1038/s41598-024-72303-4

**Published:** 2024-09-17

**Authors:** Oi-ling Siu, Kelvin F. H. Lui, Yi Huang, Ting Kin Ng, Wai Lan Victoria Yeung

**Affiliations:** 1 Department of Psychology, Lingnan University, Tuen Mun, Hong Kong, China; 2 Wofoo Joseph Lee Consulting and Counselling Psychology Research Centre, Lingnan University, Hong Kong, China

Correction to: *Scientific Reports* 10.1038/s41598-024-66974-2, published online 09 July 2024

Wai Lan Victoria Yeung was omitted from the author list in the original version of this Article.

The Author Contributions section now reads:

“Oi-ling Siu acquired the funding. All authors conceptualized the research ideas and designed the study. K. Lui and T. K. Ng analyzed the data. K. Lui drafted the manuscript. All authors reviewed, edited, and approved the final version of the manuscript.”

The original Article has been corrected.

