# Supplementary material for: An efficient, reliable and valid assessment for affective states during online learning
Source: Sci Rep. 2024 Jul 9;14:15768. doi: 10.1038/s41598-024-66974-2 (PMC11233674; doi:10.1038/s41598-024-66974-2)
Supplement: Supplementary file 1 — Supplementary Information. [file 41598_2024_66974_MOESM1_ESM.docx]

**Supplementary Materials I. A systematic review of studies examining the relationship between the five affective states and learning outcomes**

**Table S1.** Summary of the systematic review

| **Affective states** | **Relevant findings** | **Number of Studies** | **References** |
| --- | --- | --- | --- |
| Concentration | Positive association with learning outcomes | 16 | [1-16] |
| Motivation | Positive association with learning outcomes | 17 | [17-34] |
|  | No significant association | 2 | [35,36] |
| Perseverance | Positive association with learning outcomes | 14 | [37-50] |
|  | No significant association | 6 | [51-56] |
| Engagement | Positive association with learning outcomes | 25 | [57-81] |
| Self-initiative | Positive association with learning outcomes | 17 | [82-98] |
|  | No significant association | 1 | [99] |
|  | Mixed findings | 1 | [100] |

**References**

1. Hembrooke, H., & Gay, G. (2003). The laptop and the lecture: The effects of multitasking in learning environments. *Journal of computing in higher education, 15*, 46-64. <https://doi.org/10.1007/BF02940852>
2. Leigh, E., Chiu, K., & Clark, D. M. (2021). Is concentration an indirect link between social anxiety and educational achievement in adolescents?. *PloS one, 16*(5), e0249952. <https://doi.org/10.1371/journal.pone.0249952>
3. Li, X., & Yang, X. (2016). Effects of learning styles and interest on concentration and achievement of students in mobile learning. *Journal of Educational Computing Research, 54*(7), 922-945. <https://doi.org/10.1177/0735633116639953>
4. Lu, T., & Yang, X. (2018). Effects of the visual/verbal learning style on concentration and achievement in mobile learning. *EURASIA Journal of Mathematics, Science and Technology Education, 14*(5), 1719-1729. <https://doi.org/10.29333/ejmste/85110>
5. Maiztegi-Kortabarria, J., Arribas-Galarraga, S., Luis-de Cos, I., Espoz-Lazo, S., & Valdivia-Moral, P. (2024). Effect of an Active Break Intervention on Attention, Concentration, Academic Performance, and Self-Concept in Compulsory Secondary Education. *European Journal of Investigation in Health, Psychology and Education, 14*(3), 447-462. <https://doi.org/10.3390/ejihpe14030030>
6. Narsih, N., Sappaile, B. I., & Nasrullah, N. (2022). The Relationship between Learning Concentration and Student Emotional Maturity to Mathematics Learning Outcomes of Class X Students of High School. *SAINSMAT: Journal of Applied Sciences, Mathematics, and Its Education, 11*(2), 73-80. <https://doi.org/10.35877/sainsmat427>
7. Pardos, Z. A., Baker, R. S., San Pedro, M. O., Gowda, S. M., & Gowda, S. M. (2013, April). Affective states and state tests: Investigating how affect throughout the school year predicts end of year learning outcomes. In *Proceedings of the third international conference on learning analytics and knowledge* (pp. 117-124). <https://doi.org/10.1145/2460296.2460320>
8. Rodríguez-García, L., de la Cruz-Campos, J. C., Martín-Moya, R., & González-Fernández, F. T. (2022). Active Teaching Methodologies Improve Cognitive Performance and Attention-Concentration in University Students. *Education Sciences, 12*(8), 544. <https://doi.org/10.3390/educsci12080544>
9. Sadiyani, N. W. (2018). Strategy of improving student achievement in English learning through concentration approach. *International Journal of Physical Sciences and Engineering, 2*(1), 47-56.
10. Sana, F., Weston, T., & Cepeda, N. J. (2013). Laptop multitasking hinders classroom learning for both users and nearby peers. *Computers & Education, 62*, 24–31. <https://doi.org/10.1016/j.compedu.2012.10.003>
11. Saputra, D. C. E., Azhari, A., & Ma'arif, A. (2022). K-Nearest Neighbor of Beta Signal Brainwave to Accelerate Detection of Concentration on Student Learning Outcomes. *Engineering Letters, 30*(1).
12. Sati, L., & Sunarti, V. (2021). The relationship between learning concentration and learning outcomes of students at lkp hazika education center. *SPEKTRUM: Jurnal Pendidikan Luar Sekolah (PLS), 9*(4), 531-544.
13. Steinmayr, R., Ziegler, M., & Träuble, B. (2010). Do intelligence and sustained attention interact in predicting academic achievement?. *Learning and Individual differences, 20*(1), 14-18. <https://doi.org/10.1016/j.lindif.2009.10.009>
14. Van der Heijden, K. B., Vermeulen, M. C., Donjacour, C. E., Gordijn, M. C., Hamburger, H. L., Meijer, A. M., ... & Weysen, T. (2018). Chronic sleep reduction is associated with academic achievement and study concentration in higher education students. *Journal of Sleep Research, 27*(2), 165-174. <https://doi.org/10.1111/jsr.12596>
15. Xiaolin, S., Suwarsi, S., Inta, P., Fajarina, A. L., Muflih,M., Retnaningsih, L. N., & Damayanti, S. (2023). The Relationship Between Learning Concentration and Understanding Level Through the Online Learning Process*. Jurnal Keperawatan Respati Yogyakarta, 10*(2), 89-93. <http://dx.doi.org/10.35842/jkry.v10i2.741>
16. Yang, X., Li, X., & Lu, T. (2015). Using mobile phones in college classroom settings: Effects of presentation mode and interest on concentration and achievement. *Computers & Education, 88*, 292-302. <https://doi.org/10.1016/j.compedu.2015.06.007>
17. Alhadi, S., & Saputra, W. N. E. (2017, May). The relationship between learning motivation and learning outcome of junior high school students in Yogyakarta. In *1st Yogyakarta International Conference on Educational Management/Administration and Pedagogy* (YICEMAP 2017) (pp. 138-141). Atlantis Press. <https://doi.org/10.2991/yicemap-17.2017.23>
18. Ambarwati, W. (2018). Influence of Parents Attention, Emotional Intelligence and Learning Motivation to Learning Outcomes. *Journal of Education, Teaching and Learning, 3*(1), 72-81. STKIP Singkawang. Retrieved June 4, 2024 from <https://www.learntechlib.org/p/209113/>.
19. Brooker, A., Corrin, L., De Barba, P., Lodge, J., & Kennedy, G. (2018). A tale of two MOOCs: How student motivation and participation predict learning outcomes in different MOOCs. *Australasian Journal of Educational Technology, 34*(1). <https://doi.org/10.14742/ajet.3237>
20. Guswara, A. M., & Purwanto, W. (2020). The contribution of Google Classroom application and motivation to the learning outcomes of web programming. *Journal of Education Technology, 4*(4), 424-432. <https://doi.org/10.23887/jet.v4i4.29896>
21. Kormos, J., & Csizer, K. (2013). The interaction of motivation, self-regulatory strategies, and autonomous learning behavior in different learner groups. *TESOL Quarterly*, 1e25. <http://dx.doi.org/10.1002/tesq.129.>
22. Kurnianto, B., Wiyanto, W., & Haryani, S. (2019). Critical thinking skills and learning outcomes by improving motivation in the model of flipped classroom. *Journal of Primary Education, 8*(6), 282-291. <https://journal.unnes.ac.id/sju/jpe/article/view/27783>
23. Lin, M. H., Chen, H. C., & Liu, K. S. (2017). A study of the effects of digital learning on learning motivation and learning outcome. *Eurasia Journal of Mathematics, Science and Technology Education, 13*(7), 3553-3564. <https://doi.org/10.12973/eurasia.2017.00744a>
24. Liu, O. L., Bridgeman, B., & Adler, R. M. (2012). Measuring Learning Outcomes in Higher Education: Motivation Matters. *Educational Researcher, 41*(9), 352-362. <https://doi.org/10.3102/0013189X12459679>
25. Peng, R., & Fu, R. (2021). The effect of Chinese EFL students’ learning motivation on learning outcomes within a blended learning environment. *Australasian Journal of Educational Technology, 37*(6), 61-74. <https://doi.org/10.14742/ajet.6235>
26. Rahmadian, R., & Maksum, H. (2020). The Contribution of Family Environment and Learning Motivation Toward Students' Learning Outcomes in Online Learning. *Journal of Education Technology, 4*(4), 385-391. <https://doi.org/10.23887/jet.v4i4.29700>
27. Salsa, F. J., Sari, R. T., Muhar, N., & Gusmaweti, G. (2022). The relationship between motivation and learning outcomes of biology subject through distance learning. *International Journal of STEM Education for Sustainability, 2*(2), 140-147.
28. Özhan, Ş. Ç., & Kocadere, S. A. (2020). The Effects of Flow, Emotional Engagement, and Motivation on Success in a Gamified Online Learning Environment. *Journal of Educational Computing Research, 57*(8), 2006-2031.
29. Taurina, Z. (2015). Students’ motivation and learning outcomes: Significant factors in internal study quality assurance system. *International Journal for Cross-Disciplinary Subjects in Education (IJCDSE), 5*(4), 2625-2630.
30. Tella, A. (2007). The Impact of Motivation on Student’s Academic Achievement and Learning Outcomes in Mathematics among Secondary School Students in Nigeria. *Eurasia Journal of Mathematics, Science and Technology Education, 3*(2), 149-156. <https://doi.org/10.12973/ejmste/75390>
31. Tokan, M. K., & Imakulata, M. M. (2019). The effect of motivation and learning behaviour on student achievement. *South African Journal of Education, 39*(1). <https://doi.org/10.15700/saje.v39n1a1510>
32. Triarisanti, R. & Purnawarman, P. (2019). The influence of interest and motivation on college students’ language and art appreciation learning outcomes. *International Journal of Education, 11*(2), 130-135. UPI Press. Retrieved June 3, 2024 from <https://www.learntechlib.org/p/208998/>.
33. Wei, X., Saab, N., & Admiraal, W. (2023). Do learners share the same perceived learning outcomes in MOOCs? Identifying the role of motivation, perceived learning support, learning engagement, and self-regulated learning strategies. *The Internet and Higher Education, 56*, 100880. <https://doi.org/10.1016/j.iheduc.2022.100880>
34. Yen, H. C., Tuan, H. L., & Liao, C. H. (2011). Investigating the influence of motivation on students’ conceptual learning outcomes in web-based vs. classroom-based science teaching contexts. *Research in Science Education, 41*, 211-224. <https://doi.org/10.1007/s11165-009-9161-x>
35. Chen, K. C., & Jang, S. J. (2010). *Motivation in online learning: Testing a model of self-determination theory. Computers in human behavior, 26*(4), 741-752. <https://doi.org/10.1016/j.chb.2010.01.011>
36. Lin, C. H., Zhang, Y., & Zheng, B. (2017). The roles of learning strategies and motivation in online language learning: A structural equation modeling analysis. *Computers & Education, 113*, 75-85. <https://doi.org/10.1016/j.compedu.2017.05.014>
37. Alamer, A. (2021). Grit and language learning: construct validation of L2-Grit scale and its relation to later vocabulary knowledge. *Educational Psychology, 41*(5), 544-562. <https://doi.org/10.1080/01443410.2020.1867076>
38. Bettinger, E., Ludvigsen, S., Rege, M., Solli, I. F., & Yeager, D. (2018). Increasing perseverance in math: Evidence from a field experiment in Norway. *Journal of Economic Behavior & Organization, 146*, 1-15. <https://doi.org/10.1016/j.jebo.2017.11.032>
39. Duckworth, A. L., Peterson, C., Matthews, M. D., & Kelly, D. R. (2007). Grit: Perseverance and passion for long-term goals. *Journal of Personality and Social Psychology, 92*(6), 1087–1101. <https://doi.org/10.1037/0022-3514.92.6.1087>
40. Hofmeyr, H. (2021). Perseverance, passion and poverty: Examining the association between grit and reading achievement in high-poverty schools in South Africa. *International Journal of Educational Development, 83*, 102376. <https://doi.org/10.1016/j.ijedudev.2021.102376>
41. Huescar Hernandez, E., Moreno-Murcia, J. A., Cid, L., Monteiro, D., & Rodrigues, F. (2020). Passion or perseverance? The effect of perceived autonomy support and grit on academic performance in college students. *International journal of environmental research and public health, 17*(6), 2143. <https://doi.org/10.3390/ijerph17062143>
42. Janelli, M., & Lipnevich, A. A. (2021). Effects of pre-tests and feedback on performance outcomes and persistence in Massive Open Online Courses. *Computers & Education, 161*, 104076. <https://doi.org/10.1016/j.compedu.2020.104076>
43. Lee, J. S. (2022). The role of grit and classroom enjoyment in EFL learners’ willingness to communicate. *Journal of Multilingual and Multicultural Development, 43*(5), 452-468. <https://doi.org/10.1080/01434632.2020.1746319>
44. Liu, E., & Wang, J. (2021). Examining the relationship between grit and foreign language performance: Enjoyment and anxiety as mediators. *Frontiers in psychology, 12*, 666892. <https://doi.org/10.3389/fpsyg.2021.666892>
45. Mason, H. D. (2018). Grit and academic performance among first-year university students: A brief report. *Journal of Psychology in Africa, 28*(1), 66–68. <https://doi.org/10.1080/14330237.2017.1409478>
46. Muenks, K., Yang, J. S., & Wigfield, A. (2018). Associations between grit, motivation, and achievement in high school students. *Motivation Science, 4*(2), 158.
47. Nagle, C. (2021). Using Expectancy Value Theory to understand motivation, persistence, and achievement in university‐level foreign language learning. *Foreign Language Annals, 54*(4), 1238-1256. <https://doi.org/10.1111/flan.12569>
48. Tang, X., Wang, M. T., Guo, J., & Salmela-Aro, K. (2019). Building grit: The longitudinal pathways between mindset, commitment, grit, and academic outcomes. *Journal of youth and adolescence, 48*, 850-863. <https://doi.org/10.1007/s10964-019-00998-0>
49. Teimouri, Y., Plonsky, L., & Tabandeh, F. (2022). L2 grit: Passion and perseverance for second-language learning. *Language Teaching Research, 26*(5), 893-918. <https://doi.org/10.1177/1362168820921895>
50. Xu, K. M., Cunha-Harvey, A. R., King, R. B., de Koning, B. B., Paas, F., Baars, M., ... & de Groot, R. (2023). A cross-cultural investigation on perseverance, self-regulated learning, motivation, and achievement. *Compare: A Journal of Comparative and International Education, 53*(3), 361-379. <https://doi.org/10.1080/03057925.2021.1922270>
51. Guo, J., Tang, X., & Xu, K. M. (2019). Capturing the multiplicative effect of perseverance and passion: Measurement issues of combining two grit facets. *Proceedings of the National Academy of Sciences, 116*(10), 3938-3940. <https://doi.org/10.1073/pnas.1820125116>
52. Karlen, Y., Suter, F., Hirt, C., & Merki, K. M. (2019). The role of implicit theories in students' grit, achievement goals, intrinsic and extrinsic motivation, and achievement in the context of a long-term challenging task. *Learning and Individual Differences, 74*, 101757. <https://doi.org/10.1016/j.lindif.2019.101757>
53. Khajavy, G. H., MacIntyre, P. D., & Hariri, J. (2021). A closer look at grit and language mindset as predictors of foreign language achievement. *Studies in Second Language Acquisition, 43*(2), 379-402. <https://doi.org/10.1017/S0272263120000480>
54. Silvervarg, A., Haake, M., Gulz, A. (2018). Perseverance Is Crucial for Learning. “OK! but Can I Take a Break?”. In *Artificial Intelligence in Education: 19th International Conference, AIED 2018, London, UK, June 27–30, 2018, Proceedings, Part I 19* (pp. 532-544). Springer International Publishing. [https://doi.org/10.1007/978-3-319-93843-1_39](https://doi.org/10.1007/978-3-319-93843-1_39.)
55. Sudina, E., & Plonsky, L. (2021). Academic perseverance in foreign language learning: An investigation of language‐specific grit and its conceptual correlates. *The Modern Language Journal, 105*(4), 829-857. <https://doi.org/10.1111/modl.12738>
56. Usher, E. L., Li, C. R., Butz, A. R., & Rojas, J. P. (2019). Perseverant grit and self-efficacy: Are both essential for children’s academic success? *Journal of Educational Psychology, 111*(5), 877–902. <https://doi.org/10.1037/edu0000324>
57. Acosta-Gonzaga, E. (2023). The effects of self-esteem and academic engagement on university students’ performance. *Behavioral Sciences, 13*(4), 348. <https://doi.org/10.3390/bs13040348>
58. Anokye Effah, N. A., & Nkwantabisa, A. O. (2022). The influence of academic engagement on academic performance of university accounting students in Ghana. *South African Journal of Accounting Research, 36*(2), 105-122. <https://doi.org/10.1080/10291954.2021.1988204>
59. Ayala, J. C., & Manzano, G. (2018). Academic performance of first-year university students: the influence of resilience and engagement. *Higher Education Research & Development, 37*(7), 1321-1335. <https://doi.org/10.1080/07294360.2018.1502258>
60. Chi, M. T., & Wylie, R. (2014). The ICAP framework: Linking cognitive engagement to active learning outcomes. *Educational psychologist, 49*(4), 219-243. <https://doi.org/10.1080/00461520.2014.965823>
61. Delfino, A. P. (2019). Student engagement and academic performance of students of Partido State University. *Asian Journal of University Education, 15*(1), n1.
62. Fredricks, J. A., Blumenfeld, P. C., & Paris, A. H. (2004). School engagement: Potential of the concept, state of the evidence. *Review of educational research, 74*(1), 59-109. <https://doi.org/10.3102/00346543074001059>
63. Gerber, C., Mans-Kemp, N., & Schlechter, A. (2013). Investigating the moderating effect of student engagement on academic performance. *Acta Academica, 45*(4), 256-274. <https://hdl.handle.net/10520/EJC151933>
64. Gray, J. A., & DiLoreto, M. (2016). The effects of student engagement, student satisfaction, and perceived learning in online learning environments. *International Journal of Educational Leadership Preparation, 11*(1), n1.
65. Gunuc, S. (2014). The relationships between student engagement and their academic achievement. *International Journal on New Trends in Education and their implications, 5*(4), 216-231.
66. Halm, D. S. (2015). The impact of engagement on student learning. *International Journal of Education and Social Science, 2*(2), 22-33.
67. Lei, H., Cui, Y., & Zhou, W. (2018). Relationships between student engagement and academic achievement: A meta-analysis. *Social Behavior and Personality: an international journal, 46*(3), 517-528. <https://doi.org/10.2224/sbp.7054>
68. Lin, L. C., Hung, I. C., Kinshuk, & Chen, N. S. (2019). The impact of student engagement on learning outcomes in a cyber-flipped course. *Educational Technology Research and Development, 67*, 1573-1591. <https://doi.org/10.1007/s11423-019-09698-9>
69. Maamin, M., Maat, S. M., & H. Iksan, Z. (2021). The influence of student engagement on mathematical achievement among secondary school students. *Mathematics, 10*(1), 41. <https://doi.org/10.3390/math10010041>
70. Mo, Y., & Singh, K. (2008). Parents’ relationships and involvement: Effects on students’ school engagement and performance. *RMLE online, 31*(10), 1-11. <https://doi.org/10.1080/19404476.2008.11462053>
71. Pike, G. R., Smart, J. C., & Ethington, C. A. (2012). The mediating effects of student engagement on the relationships between academic disciplines and learning outcomes: An extension of Holland’s theory. *Research in Higher Education, 53*, 550-575. <https://doi.org/10.1007/s11162-011-9239-y>
72. Reeve, J. (2013). How students create motivationally supportive learning environments for themselves: *The concept of agentic engagement. Journal of educational psychology, 105*(3), 579.
73. Reeve, J., & Tseng, C. M. (2011). Agency as a fourth aspect of students’ engagement during learning activities. *Contemporary educational psychology, 36*(4), 257-267. <https://doi.org/10.1016/j.cedpsych.2011.05.002>
74. Rodgers, T. (2008). Student engagement in the e-learning process and the impact on their grades. *International Journal of Cyber Society and Education, 1*(2), 143-156. <https://www.learntechlib.org/p/209167/>.
75. Sagayadevan, V., & Jeyaraj, S. (2012). The role of Emotional Engagement in Lecturer-Student interaction and the Impact on Academic Outcomes of Student Achievement and Learning. *Journal of the Scholarship of Teaching and Learning, 12*(3), 1-30.
76. Saucier, D. A., Miller, S. S., Jones, T. L., & Martens, A. L. (2022). Trickle down Engagement: Effects of Perceived Teacher and Student Engagement on Learning Outcomes. *International Journal of Teaching and Learning in Higher Education, 33*(2), 168-179.
77. Shernoff, D. J., Ruzek, E. A., & Sinha, S. (2017). The influence of the high school classroom environment on learning as mediated by student engagement. *School psychology international, 38*(2), 201-218. <https://doi.org/10.1177/0143034316666413>
78. Singh, A. K., Srivastava, S., & Singh, D. (2015). Student engagement as the predictor of direct and indirect learning outcomes in the management education context. *Metamorphosis, 14*(2), 20-29. <https://doi.org/10.1177/0972622520150204>
79. Summerlee, A., & Murray, J. (2010). The Impact of Enquiry-Based Learning on Academic Performance and Student Engagement. *Canadian Journal of Higher Education, 40*(2), 78-94.
80. Wong, L. (2013). Student Engagement with Online Resources and Its Impact on Learning Outcomes. In *E. Cohen & E. Boyd (Eds.), Proceedings of Proceedings of the Informing Science and Information Technology Education Conference 2013* (pp. 129-146). Informing Science Institute. Retrieved June 6, 2024 from <https://www.learntechlib.org/p/114687/>.
81. Wonglorsaichon, B., Wongwanich, S., & Wiratchai, N. (2014). The influence of students school engagement on learning achievement: A structural equation modeling analysis. *Procedia-Social and Behavioral Sciences, 116*, 1748-1755. <https://doi.org/10.1016/j.sbspro.2014.01.467>
82. Abd-El-Fattah, S. M. (2010). Garrison’s Model of Self-Directed Learning: Preliminary Validation and Relationship to Academic Achievement. *The Spanish Journal of Psychology, 13*(2), 586–596. [https://doi.org/10.1017/S1138741600002262](https://doi.org/10.1016/j.sbspro.2014.01.467)
83. Abdulghani, H., Almndeel, N., Almutawa, A., Aldhahri, R., Alzeheary, M., Ahmad, T., ... & Khamis, N. (2019). The validity of the self-directed learning readiness instrument with the academic achievement among the Saudi medical students. *International Journal of Medical Science and Public Health, 9*(1), 1-7.
84. Alotaibi, K. N. (2016). The learning environment as a mediating variable between self-directed learning readiness and academic performance of a sample of Saudi nursing and medical emergency students. *Nurse education today, 36*, 249-254. <https://doi.org/10.1016/j.nedt.2015.11.003>
85. Avdal, E. Ü. (2013). The effect of self-directed learning abilities of student nurses on success in Turkey. *Nurse education today, 33*(8), 838-841. <https://doi.org/10.1016/j.nedt.2012.02.006>
86. Cazan, A. M., & Schiopca, B. A. (2014). Self-directed learning, personality traits and academic achievement. *Procedia-Social and Behavioral Sciences, 127*, 640-644. <https://doi.org/10.1016/j.sbspro.2014.03.327>
87. Chou, P. N. (2012). Effect of students’ self-directed learning abilities on online learning outcomes: Two exploratory experiments in electronic engineering. *International Journal of Humanities and Social Science, 2*(6), 172-179.
88. Chou, P. N. (2012). The relationship between engineering students' self-directed learning abilities and online learning performances: A pilot study. Contemporary Issues in *Education Research, 5*(1), 33-38.
89. Kan’an, A., & Osman, K. (2015). The relationship between self-directed learning skills and science achievement among Qatari students. *Creative education, 6*(08), 790.
90. Khalid, M., Bashir, S., & Amin, H. (2020). Relationship between Self-Directed Learning (SDL) and Academic Achievement of University Students: A Case of Online Distance Learning and Traditional Universities. *Bulletin of Education and Research, 42*(2), 131-148.
91. Khiat, H. (2017). Academic performance and the practice of self-directed learning: The adult student perspective. *Journal of further and Higher Education, 41*(1), 44-59. <https://doi.org/10.1080/0309877X.2015.1062849>
92. Lounsbury, J. W., Levy, J. J., Park, S. H., Gibson, L. W., & Smith, R. (2009). An investigation of the construct validity of the personality trait of self-directed learning. *Learning and Individual Differences, 19*(4), 411-418. <https://doi.org/10.1016/j.lindif.2009.03.001>
93. Nordin, N., Abd Halim, N., & Malik, M. (2016). Assessing readiness for self-directed learning among college students in the provision of higher learning institution. *Environment-Behaviour Proceedings Journal, 1*(3), 91-101. <https://doi.org/10.21834/e-bpj.v1i3.352>
94. Saeid, N., & Eslaminejad, T. (2017). Relationship between Student's Self-Directed-Learning Readiness and Academic Self-Efficacy and Achievement Motivation in Students. *International education studies, 10*(1), 225-232.
95. Stewart, R. A. (2007). Investigating the link between self directed learning readiness and project-based learning outcomes: the case of international Masters students in an engineering management course. *European Journal of Engineering Education, 32*(4), 453-465. <https://doi.org/10.1080/03043790701337197>
96. Tekkol, İ. A., & Demirel, M. (2018). An investigation of self-directed learning skills of undergraduate students. *Frontiers in psychology, 9*, 410879. <https://doi.org/10.3389/fpsyg.2018.02324>
97. Vashe, A., Devi, V., Rao, R., Abraham, R. R., & Pallath, V. (2013, December). Link between self-directed learning readiness and academic performance of medical students. In *2013 IEEE International Conference in MOOC, Innovation and Technology in Education* (MITE) (pp. 130-133).
98. Wolters, C. A., & Hussain, M. (2015). Investigating grit and its relations with college students’ self-regulated learning and academic achievement. *Metacognition and Learning, 10*, 293-311. <https://doi.org/10.1007/s11409-014-9128-9>
99. Deyo, Z. M., Huynh, D., Rochester, C., Sturpe, D. A., & Kiser, K. (2011). Readiness for self-directed learning and academic performance in an abilities laboratory course. *American Journal of Pharmaceutical Education, 75*(2), 25. <https://doi.org/10.5688/ajpe75225>
100. Chou, P. N., & Chen, W. F. (2008). Exploratory study of the relationship between self-directed learning and academic performance in a web-based learning environment. *Online Journal of Distance Learning Administration, 11*(1), 15-26.

**Supplementary Materials II. Affective States for Online Learning Scale**

**English Version**

Please select the appropriate number (from 1 = strongly disagree to 6 = strongly agree) to indicate to what extent you agree or disagree with the following statements.

|  | Strongly disagree | Disagree | Slightly Disagree | Slightly agree | Agree | Strongly Agree |
| --- | --- | --- | --- | --- | --- | --- |
| **Concentration** | | | | | | |
| During the past half hour, I concentrated on learning. | 1 | 2 | 3 | 4 | 5 | 6 |
| During the past half hour, I did not shift my attention to tasks other than learning. | 1 | 2 | 3 | 4 | 5 | 6 |
| During the past half hour, my learning was not interrupted. | 1 | 2 | 3 | 4 | 5 | 6 |
| **Motivation** | | | | | | |
| During the past half hour, I enjoy learning materials that are completely new to me. | 1 | 2 | 3 | 4 | 5 | 6 |
| During the past half hour, what matters most to me is enjoying learning. | 1 | 2 | 3 | 4 | 5 | 6 |
| During the past half hour, it is important for me to be able to learn what I most enjoy. | 1 | 2 | 3 | 4 | 5 | 6 |
| **Perseverance** | | | | | | |
| During the past half hour, I finish the learning I begin. | 1 | 2 | 3 | 4 | 5 | 6 |
| During the past half hour, I keep at my learning until I am done with it. | 1 | 2 | 3 | 4 | 5 | 6 |
| During the past half hour, once I make a learning plan, I stick to it. | 1 | 2 | 3 | 4 | 5 | 6 |
| **Engagement** | | | | | | |
| During the past half hour, I feel happy when I am studying intensely. | 1 | 2 | 3 | 4 | 5 | 6 |
| During the past half four, I am immersed in my studies. | 1 | 2 | 3 | 4 | 5 | 6 |
| During the past half hour, I get carried away when I am studying. | 1 | 2 | 3 | 4 | 5 | 6 |
| **Self-initiative** | | | | | | |
| During the past half hour, I want to learn new information. | 1 | 2 | 3 | 4 | 5 | 6 |
| During the past half hour, I enjoy learning new information. | 1 | 2 | 3 | 4 | 5 | 6 |
| During the past half hour, I have a need to learn. | 1 | 2 | 3 | 4 | 5 | 6 |

**Chinese Version**

請選擇適當的數字(從1 = 非常不同意至6 = 非常同意)去表達你對下列陳述的同意或不同意程度。

|  | 非常不同意 | 不同意 | 少許不同意 | 少許同意 | 同意 | 非常同意 |
| --- | --- | --- | --- | --- | --- | --- |
| **專注** | | | | | | |
| 在過去的半小時，我只專注於學習。 | 1 | 2 | 3 | 4 | 5 | 6 |
| 在過去的半小時，我沒有將注意力分散去學習以外的其他事情。 | 1 | 2 | 3 | 4 | 5 | 6 |
| 在過去的半小時，我沒有受到其他事情的干擾。 | 1 | 2 | 3 | 4 | 5 | 6 |
| **動力** | | | | | | |
| 在過去的半小時，我喜歡在學習全新的教材中找到樂趣。 | 1 | 2 | 3 | 4 | 5 | 6 |
| 在過去的半小時，能在學習的過程中得到享受對我來說至關重要。 | 1 | 2 | 3 | 4 | 5 | 6 |
| 在過去的半小時，能夠學到感興趣的事物對我來說至關重要。 | 1 | 2 | 3 | 4 | 5 | 6 |
| **毅力** | | | | | | |
| 在過去的半小時，只要學習開始，我就會完成它。 | 1 | 2 | 3 | 4 | 5 | 6 |
| 在過去的半小時，我堅持學習直到完成爲止。 | 1 | 2 | 3 | 4 | 5 | 6 |
| 在過去的半小時，一旦我制定了學習計劃，我就會按計劃進行。 | 1 | 2 | 3 | 4 | 5 | 6 |
| **投入** | | | | | | |
| 在過去的半小時，當我緊張學習時，我會感到快樂。 | 1 | 2 | 3 | 4 | 5 | 6 |
| 在過去的半小時，我連續學習很長時間。 | 1 | 2 | 3 | 4 | 5 | 6 |
| 在過去的半小時，我在學習時達到忘我的境界。 | 1 | 2 | 3 | 4 | 5 | 6 |
| **自我主動** | | | | | | |
| 在過去的半小時，我想要學習新信息。 | 1 | 2 | 3 | 4 | 5 | 6 |
| 在過去的半小時，我享受學習新信息。 | 1 | 2 | 3 | 4 | 5 | 6 |
| 在過去的半小時，我有學習的需要。 | 1 | 2 | 3 | 4 | 5 | 6 |
